# Incorporation of Biosolids as Water Replacement in a Two-Step Renewable Hydrocarbon Process: Hydrolysis of Brown Grease with Biosolids

**DOI:** 10.1007/s12649-019-00897-2

**Published:** 2019-12-10

**Authors:** Lin Xia, Michael Chae, Justice Asomaning, Mehdi Omidghane, Chengyong Zhu, David C. Bressler

**Affiliations:** Department of Agricultural, Food and Nutritional Science, University of Alberta, Edmonton, T6G 2P5 Canada

**Keywords:** Biosolids, Hydrolysis, Brown grease, Fatty acids, Renewable hydrocarbons, Drop-in fuels

## Abstract

**Abstract:**

The accumulating volumes of biosolids in lagoons worldwide have intensified the need to develop innovative wastewater treatment strategies. Here, we provide proof-of-concept for the incorporation of biosolids into the hydrolysis step of a two-step thermal conversion of lipids for production of renewable hydrocarbons, which can be utilized as renewable fuels. Brown grease was hydrolysed with biosolids or water at 260–280 °C for 60 min at a mass ratio of 1:1 feed to water or biosolids. The feedstock and products were characterized using various analytical techniques to compare the performance of biosolids to water. The results indicated that there was no significant difference in the degree of hydrolysis of brown grease when biosolids was used as water replacement. The fatty acids composition after hydrolysis when biosolids was used as a water replacement also remained largely unchanged. Hydrolysis of brown grease with biosolids could be achieved at pH ranging from 3.3 to 8.9, and at a lower than previously established temperature. Significantly, the rapid settling of solid material in biosolids observed after thermal hydrolysis of brown grease may reduce the necessity of biosolids settling lagoons. Thus, incorporation of biosolids into a lipid hydrolysis-pyrolysis process may simultaneously benefit the biofuel and waste management sectors.

**Graphic Abstract:**

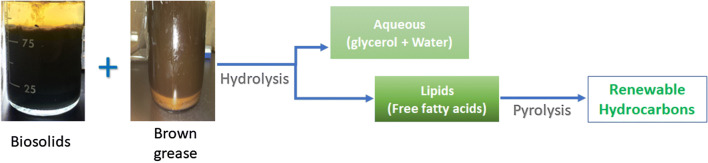

## Statement of Novelty

Biosolids storage lagoons are part of municipal wastewater treatment plants worldwide. Due to concerns of potential pathogens and toxins, biosolids have limited application and there is currently an urgent need to find a solution to the ever-increasing accumulation of biosolids. Here we provide a novel two-step thermal process as proof-of-concept that aims at solving the biosolids problem and producing drop-in biofuels. In the first step, brown grease (a low-quality lipid feedstock) is hydrolyzed with biosolids and the resulting fatty acids are further thermally processed to renewable hydrocarbons that can be used as drop-in fuels. The hydrolysis conditions employed are above sterilization conditions and has been shown to improve biosolids settling.

## Introduction

Biosolids are the residues of wastewater treatment technologies and are typically stored in large settling lagoons that facilitate gradual thickening over a 1–4 year period [1]. In the United States alone, more than 8 million dry tonnes of biosolids are produced each year [2]. Currently, thickened biosolids have few applications and are most commonly used as a fertilizer, providing both water and nutrients for plant growth [3]. However, there is a growing concern that land application of biosolids may introduce various pathogens and toxins into the environment [4–6]. The limited end uses for biosolids combined with the anticipated increase in biosolids production as the global population continues to climb, along with the inaccessibility of land for construction of new lagoons, have compelled municipalities to seek out new and innovative strategies to dispose of biosolids.

The key issues surrounding the disposal of biosolids are their extremely high water content (93–99% water; [7]) and their incredibly slow rates of settling [1], which make the removal of solid materials inefficient and/or costly. Chae et al. described how thermal hydrolysis at 280 °C for 1 h dramatically increased the settling rates of biosolids and thus may serve as an alternative to using biosolids lagoons [8]. While some municipalities may have no other options than to employ an expensive process for the disposal of biosolids, the high capital and operational costs associated with thermal hydrolysis may be prohibitive in other towns and cities. An alternative suggested by Chae et al. was to incorporate biosolids into existing high temperature processes, such as lipid pyrolysis [8].

For instance, a two-step thermal conversion technology that can convert various lipid feedstocks to renewable fuels and chemicals has been reported by Asomaning et al. [9]. In the first step of the process, lipid feedstocks are subjected to thermal hydrolysis, which converts acylglycerol based lipids into free fatty acids and glycerol. In the second step, the free fatty acids produced in the first step are subjected to pyrolysis where the fatty acids undergo deoxygenation and thermal cracking to produce renewable hydrocarbon [10]. Several lipid feedstocks have been examined for the production of free fatty acids through thermal hydrolysis including, oleaginous yeast and microalgae [11, 12], plant oils [13, 14], and waste yellow and brown greases [9]. The ability to accommodate a wide range of lipid feedstocks makes thermal hydrolysis an extremely promising technology for biofuels production.

The aim of this study was to examine whether biosolids that consist mostly of water could serve as a water replacement during hydrolysis of lipid feedstocks such as brown grease. In this manner, it may be possible to reduce costs associated with water usage during production of renewable fuels and chemicals, while at the same time provide a strategy for disposal of biosolids. To this end, brown grease was thermally hydrolyzed with biosolids under different pH and temperatures and the degree of hydrolysis was assessed to establish feasibility of incorporating biosolids into lipid pyrolysis.

## Materials and Methods

### Feedstocks

The biosolids used in these studies were obtained from a biosolids lagoon at a wastewater treatment facility in Edmonton, Canada. Thus, the biosolids samples received were the residues after clarification, digestion, and stabilization, and were being stored in lagoons for further thickening and settlement. According to the supplier, the biosolids contained approximately 3.5% solids. Due to safety concerns, biosolids were sterilized via autoclaving at 121 °C, 20 min, and 15 psig (~ 207 kPa) unless they were to be subjected to thermal hydrolysis. Autoclaving is a comparatively mild thermal treatment and is not anticipated to result in substantial changes in biosolids composition. Brown grease samples were generously provided by a large Canadian rendering company.

### Solvents and Analytical Standards

Standards for gas chromatography (oleic acid, ≥ 99%), high performance liquid chromatography (nonadecanoic acid methyl ester, 99%), and thin layer chromatography (glyceryl trioleate, ≥ 99%; dioleoylglycerol, ≥ 99%; 1-oleoyl-rac-glycerol, ≥ 99%; and oleic acid, ≥ 99%) were purchased from Sigma-Aldrich (St. Louis, MO, USA). For gas chromatography, derivatization of all fatty acids (both esterified and free fatty acids) was achieved using acetyl chloride (≥ 99%, Sigma-Aldrich (St. Louis, MO, USA)), while derivitiation of free fatty acids alone was accomplished using diazomethane prepared from diazald (TLC Pharmaceutical Standards Ltd., Aurora, ON, Canada) and a Diazald® kit from Sigma-Aldrich (St. Louis, MO, USA). For thin layer chromatography, iodine (≥ 99.99%) and diethyl ether (≥ 98%) were also purchased from Sigma-Aldrich (St. Louis, MO, USA). The solvents used for analysis, methanol (HPLC grade, > 99.9%), toluene (HPLC grade, > 99.9%), hexane (HPLC grade, > 99.9%), acetic acid (> 99.85%), along with the *o*-phosphoric acid (85%) used for acidification, were all obtained from Fisher Scientific (Fairlawn, NJ, USA). Nitrogen gas (99.998%) was obtained from Praxair (Mississauga, ON, Canada).

### Thermal Hydrolysis

#### Thermal Hydrolysis of Biosolids

Thermal hydrolysis of biosolids was conducted to provide insight into the distribution of compounds and elements after hydrolysis. It also provided a baseline by which to compare the hydrolysis of brown grease with biosolids and how it could impact the composition of the resulting free fatty acids. A batch 5.5 L reactor (Model 4580, Parr Instrument Company, Moline, IL, USA) was used at 280 °C or 260 °C for 60 min with constant stirring at 200 rpm. The use of the large batch reactor for the thermal hydrolysis of biosolids was because of the relatively low amounts of dry matter in the biosolids, a significant portion of which was inorganic (ash). This was to allow enough sample to be collected for analysis. The reactor was purged with nitrogen at 500 psi (3.44 MPa) to provide an oxygen free atmosphere after which the reactor was set to an initial pressure of 100 psi (689 kPa) using nitrogen gas. The reactor pressure stabilized at 1200–1300 psi (8.27–8.96 MPa) when the set temperature was reached. The starting point for the reaction was defined as the time when the set temperature was reached. When the reaction was complete, the heater was turned off and the reactor was cooled to room temperature using an external cooling system (VWR, Radnor, PA, USA) set to − 20 °C.

#### Thermal Hydrolysis of Brown Grease with Water or Biosolids

Hydrolysis of brown grease was conducted in lab-scale 15 mL batch microreactors constructed with stainless steel Swagelok fittings and tubing (0.75 inches), which were heated in a Techne SBS-4 fluidized bed sand bath with a TC-D8 controller (Burlington, NJ, USA). The vessel had an internal volume of 15 ml and a pressure rating of 3,300 psi. Reactions were carried out at 280 °C and a 1:1 (w/w) liquid to lipid (water or biosolids) ratio. For the biosolids hydrolysis runs, the effect of biosolids pH was determined by adjusting the initial pH to 6.2 (similar to the pH of the distilled water that was used) and pH 3.3 using phosphoric acid. Phosphoric acid was selected because other mineral acids such as hydrochloric acid and sulfuric acid are corrosive to the stainless-steel reactor. Samples were loaded into a clean and dry microreactor; after the reactor was closed, it was purged with nitrogen to provide an inert atmosphere and pressurized to an initial pressure of 500 psi (3.44 MPa). The reactor was placed into a sand bath preheated to the desired temperature. The reaction was conducted with agitation for 1 h. The vessel was then removed from the sand bath and immediately cooled with water to room temperature.

### Lipid Extraction

#### Extraction of Lipids from Biosolids Using Hexane

Since the lipid content of the biosolids used in these studies was very low, hexane was used to extract organic substances such as free fatty acids, acylglycerol-based lipids (triacylglycerols, diacylglycerols, and monoacylglycerols), as well as other hexane soluble compounds from autoclaved and hydrolyzed biosolids. The original biosolids were acidified to pH 3 using phosphoric acid after autoclaving in order to protonate the free fatty acids present in the aqueous phase, which is necessary for hexane extraction. Autoclaved or hydrolyzed biosolids were homogenized and then extracted with hexane at a ratio of 1:1 (v/v) three times. After extraction, the hexane layer was filtered through #1 Whatman filter paper (Whatman, Maidstone, Kent, UK) to remove traces of cell debris and small solid particles suspended in the hexane extracts. A Buchi R-205 rotary evaporator (Büchi Labortechnik AG, Flawil, Switzerland) was then used to remove most of the hexane at 40 °C under 325 mmHg of vacuum, and extracts were concentrated down to ~ 10 mL. The concentrated organic phase was transferred to a volumetric flask and brought to a constant volume of 25 mL using *n*-hexane. After this, 10 mL was transferred to a pre-weighed glass vial, and the solvent was removed at 25 °C under N_2_ using an analytical evaporator system (Glas-col, Terre Haute, IN, USA). The recovered hexane extract was weighed for calculations. The extracts and the rest of solution were stored at 4 °C until further analysis was performed.

#### Extraction of Lipids from Brown Grease Hydrolysates

Following thermal hydrolysis of brown grease with water or biosolids, the contents of the reaction vessel were transferred by carefully decanting to a 15 mL centrifuge tube. To facilitate separation, the samples were centrifuged at 8648×*g* for 5 min in an accuSpin 400 centrifuge (Fisher Scientific, Osterode, Germany). After this, the top layer (lipid phase) was carefully transferred to a new centrifuge tube and stored at 4 °C until required for further analyses.

### Characterization and Analytical Determinations

The moisture content was determined gravimetrically by drying samples in a freeze dryer (VirTis Ultra 35L, SP Scientific, Stone Ridge, NY, USA). Samples were loaded to pre-weighed 50 mL plastic conical centrifuge tubes (Fisher Scientific, Fairlawn, NJ, USA), frozen at − 80 °C, and then freeze-dried for 72 h. The samples were weighed and masses were recorded. Then, they were re-dried for an additional 2 h. The weight check procedure was performed several times until no further weight changes were observed. Water content was calculated by the mass difference between samples before and after freeze drying.

The ash content was determined gravimetrically by burning the sample in a muffle furnace at 550 °C for 1 h, followed by cooling down in desiccator following ASTM D5347-95 [15]. The weight was then recorded and the sample was reheated in the muffle oven at 550 °C for another 1 h. The cool down and weight check procedures were repeated several times until there was no change of weight.

The CHNS analysis was conducted by the Department of Chemistry, University of Alberta using a Thermo Flash 2000 Elemental Analyzer (Thermo Fisher, Milan, Italy). The metal content was determined by inductively coupled plasma mass spectrometry (ICP-MS; Elan 6000, Perkin-Elmer SCIEX, Toronto, ON, Canada) in the Department of Earth and Atmospheric Sciences, University of Alberta.

#### Thin Layer Chromatography (TLC)

A Whatman aluminum silica TLC plate (Maidstone, Kent, UK) with hexane:diethyl ether:acetic acid (80:20:1) as the mobile phase was used for qualitative analysis of the lipid classes in select sample. A 6 mg/mL sample or standard in hexane was prepared. For analysis, 2 μL of the 6 mg/mL sample or standard solution were spotted on the marked line of the plate and developed in a closed TLC chamber with the sample spot well above the level of the mobile phase. The free fatty acids, triacylglycerols, diacylglycerols, and monoacylglycerols will be separated according to their polarity, with the least polar compounds traveling furthest up the plate than the more polar compounds. Visualization of the spots was done in an iodine chamber.

#### High Performance Liquid Chromatography (HPLC)

Identification and quantification of various lipid classes were studied using a HPLC size-exclusion chromatography system consisting of an Agilent 1200 series binary pump, a high-performance autosampler, and an evaporative light scattering detector (ELSD) (Agilent Technologies, Santa Clara, CA, USA). In these experiments, a 100 Å Phenogel size exclusion column (300 mm × 7.8 mm internal diameter × 5 m) protected with a SecurityGuard C18 guard cartridge system (Phenomenex, Terrence, CA, USA), was used to analyze the triacylglycerol, diacylglycerol, monoacylglycerol, and free fatty acid composition of various samples. The detector temperature was set at 40 °C, and N_2_ gas was set as 3.5 bars (350 kPa). Toluene containing 0.25% acetic acid was chosen as the mobile phase as it allows for better resolution of the lipid classes as described by Kittirattanapiboon et al. [16]. The flow rate of the mobile phase was 1.0 mL/min, the concentration of samples was 3.5 mg/mL, and all the standards and samples were prepared by dissolving in toluene (> 99.9%). Quantification of the lipid classes were determined from external calibration standard of triolein, diolein, monoolein, and oleic acid.

#### Gas Chromatography (GC)

Detailed profiles of free fatty acids, hydrocarbons, and other organic compounds were obtained through GC combined with mass spectrometer or quantified by GC with a flame ionization detector (FID). Diazomethane was used as an esterification reagent to methylate free fatty acids since this method was quick and simple and can selectively derivatize free fatty acids. Conversely, to determine the total amount of fatty acids (free fatty acids and esterified/acylglycerol-based fatty acids), acetyl chloride (10% in methanol) was used as methylation agent at a reaction temperature of 80 °C for 2 h [17].

Analysis of derivatives was conducted on an Agilent 6890N gas chromatograph equipped with a FID and an HP 7683 autosampler. Helium was used as the carrier gas at a constant flow rate of 1 mL/min. Separation of components was performed on a 30 m × 0.32 mm (internal diameter) HP-5 ms capillary column with a 0.25 μm film thickness (Agilent Technologies, Santa Clara, CA, USA). The injector and detector temperature were set at 300 °C and 350 °C, respectively. The following oven temperature program was used: 0.1 min hold at 35 °C, ramp to 280 °C at 10 °C per minute, hold at 280 °C for an additional 5.4 min, for a total run time of 30 min. A 1:40 split injection ratio was used, and the injection volume was 1 μL.

GC with mass spectrometric detection (GC–MS) analysis was also employed using a similar column and conditions as GC-FID (described above) and performed on an Agilent GC 6890N coupled to an Agilent 5975B EI/CI MS instrument operated in electron ionization (EI) mode (Agilent Technologies, Santa Clara, CA, USA). The temperature of the GC–MS interface was kept constant at 320 °C.

### Degree of Hydrolysis

The degree of hydrolysis was used to evaluate the extent of hydrolysis and to compare the effectiveness of using biosolids as water replacement during the hydrolysis step. The fatty acids data acquired through GC-FID and using the following equation was used to determine the degree of hydrolysis:

1 $$ {\text{Degree of }}\;{\text{hydrolysis}} = \frac{{{\text{FFA\% }}_{{\left( {{\text{Hydrolysate}}} \right)}} - {\text{FFA\% }}_{{\left( {\text{Brown grease}} \right)}} }}{{{\text{TFA}}\%_{{\left( {\text{Brown grease}} \right)}} - {\text{FFA}}\%_{{\left( {\text{Brown grease}} \right)}} }} \times 100 $$

where **FFA% **_**(Hydrolysate)**_ = The amount of free fatty acids in the hydrolysate as determined through derivatization with diazomethane. **FFA% **_**(Brown Grease)**_ = The amount of free fatty acids in the original brown grease as determined through derivatization with diazomethane. **TFA% **_**(Brown Grease)**_ = The total amount of fatty acids (both free and esterified fatty acids) in the original brown grease as determined through derivatization with acetyl chloride.

### Statistical Analysis

The statistical analysis of data was performed using one-way ANOVA with the mean comparison by Tukey test, or* t* test where appropriate using GraphPad Prism 6 software, (Graphpad Software Inc. La Jolla, CA USA), based on a confidence level of 95%.

## Results and Discussion

### Characterization of Biosolids

Although the main focus of this research was to determine whether biosolids could be incorporated into lipid pyrolysis as a water replacement and a source of additional lipid material, we first examined the hydrolysis of biosolids themselves. These experiments served as a baseline and provided some insight into subsequent hydrolysis experiments with brown grease. The biosolids used in these experiments were acquired from a lagoon at a wastewater treatment facility in which the biosolids settle naturally after removal from an anaerobic digester. These biosolids consisted mostly of water (96.8 ± 0.1%), with a small amount of ash (1.0 ± 0.1%) and virtually no free fatty acids (0.008 ± 0.001%). Following thermal hydrolysis of the biosolids at 280 °C for 1 h, there was no significant difference in terms of ash content, but there was a small decrease in the amount of water (from 96.8 ± 0.1 to 96.5 ± 0.1%), likely stemming from the consumption of water molecules during hydrolysis of lipids and other organic molecules or alternatively evaporative loss during handling. There was also a significant increase in the amount of free fatty acids (from 0.008 ± 0.001 to 0.03 ± 0.01%). This suggests the presence of a significant amount of acylglycerols compared to free fatty acids in the biosolids. The acylglycerol under the conditions used in this study would undergo hydrolysis releasing free fatty acids, which was responsible for the observed increase in free fatty acids.

### Characterization of the Hexane Extract from Biosolids

In lipid pyrolysis, free fatty acids from a wide variety of feedstocks undergo decarboxylation and decarbonylation leading to the formation of hydrocarbons that can be used as renewable fuels [10]. The feedstock for lipid pyrolysis used by Asomaning et al. [10] was a lipid-rich fraction recovered from brown grease hydrolysis. Since any materials contained within this lipid-rich phase may influence the quality of the renewable fuel generated through lipid pyrolysis, we conducted compositional analyses on the hexane extract recovered from biosolids, either before or after thermal hydrolysis, to acquire insight into the effects of thermal hydrolysis on biosolids.

#### Elemental and Metal Analyses

**Fig. 1:**
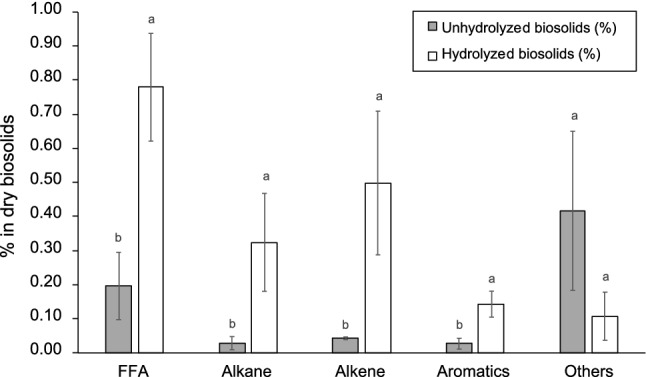
Organic components in biosolids (based on dry mass) before and after hydrolysis at 280 °C for 1 h in a 5.5 L reactor. Data shows mean ± standard deviation with n = 3. The statistical analysis was done using one-way ANOVA with mean comparison by Tukey test based on a 95% confidence level

The elemental composition of the hexane extract of hydrolyzed biosolids was as follows: 78.6 ± 1.6% carbon, 11.0 ± 0.2% hydrogen, 1.2 ± 0.5% nitrogen, 1.9 ± 0.6% sulphur, and 6.1 ± 0.9% oxygen. The relatively low oxygen content of the hexane extract of hydrolyzed biosolids is important to note. This indicates the possibility of low lipid levels, in particular, acylglycerols and free fatty acids and/or the presence of high amounts of oxygen free hydrocarbon species. The oxygen content of hydrolyzed acylglycerol-based lipids has been reported to range from 12 to 15% depending on the source of the feedstock [9]. Figure 1 shows the different classes of compounds present in the hexane extract of the hydrolyzed biosolids. Indeed, Fig. 1 shows that the combined amounts of oxygen free hydrocarbons (alkanes, alkenes and aromatics) was similar to the amount of free fatty acids after hydrolysis. This is an important observation as the development of drop-in renewable alternatives to petroleum-derived liquid fuels requires the removal of oxygen as a necessary step.

The presence of significant amounts of nitrogen and sulphur in the hexane extract of the hydrolyzed biosolids is also important to consider as they may end up in the fuel produced after pyrolysis. Thus, the presence of these species, especially sulphur in the pyrolysis product, need to be monitored to ensure that they meet local and international fuel standards and regulations. However, as stated in section "Characterization of Biosolids", the amount of organic material in the biosolids is less than 2% and hence this sulphur is not expected to be a significant contributor to the overall mass and sulphur content when used as a water replacement. This implies the amount of nitrogen and sulphur that will end up in the lipid fraction when biosolids is used as water replacement for the hydrolysis of a much larger volumetric lipid source, may be significantly diluted from what is reported for the hexane extract of hydrolyzed biosolids.

The metal content of the hexane extract recovered from biosolids or thermally hydrolyzed biosolids was determined using ICP-MS to ascertain whether thermal hydrolysis impacted distribution of metals (Table 1). The most abundant metals in both samples were Fe, Ca, Mg, and Al. After hydrolysis, metals such as Mg, Ca, Cr, Mn, Se, Sn, and Pb significantly decreased in the hexane extract; the only metal to show an increased level after hydrolysis was As. Since the presence of heavy metals in fuels can damage car engine and emission systems, it is encouraging that hydrolysis seems to result in the removal of some metals from the organic phase. However, it remains to be seen whether any metals in the organic phase would be found in the gasoline and/or diesel cuts obtained after pyrolysis and subsequent distillation of such lipid-rich feedstocks.

**Table 1:** Elemental analysis of biosolids hexane extract by ICP-MS

Metal	Biosolids hexane extract	
	Before hydrolysis (ppm)	After hydrolysis (ppm)
B	15 ± 9	36 ± 31
Na	65 ± 26	59 ± 55
Mg*	118 ± 36	32 ± 22
Al	113 ± 5	73 ± 54
K	BDL	44 ± 45
Ca*	580 ± 197	54 ± 5
V	4 ± 3	1.7 ± 0.7
Cr*	12 ± 2	3 ± 2
Fe	285 ± 193	169 ± 56
Mn*	5.7 ± 1.3	1.1 ± 0.7
Co	0.9 ± 0.8	0.4 ± 0.2
Ni	5 ± 3	12 ± 6
Cu	20 ± 6	23 ± 3
Zn	10 ± 8	10 ± 10
As*	1.2 ± 0.2	6 ± 3
Se*	9 ± 4	1.1 ± 0.1
Sr	5 ± 3	0.9 ± 0.7
Y	0.10 ± 0.02	BDL
Zr	33 ± 21	2.4 ± 1.5
Nb	0.3 ± 0.1	0.3 ± 0.2
Ag	1 ± 0.4	1.2 ± 0.9
Cd	0.12 ± 0.01	BDL
Sn*	6.1 ± 1.5	0.6 ± 0.1
Sb	0.12 ± 0.06	0.7 ± 0.3
Ba	3.9 ± 0.7	1.9 ± 1.4
Ta	0.12 ± 0.02	0.4 ± 0.3
Os	0.2 ± 0.1	0.5 ± 0.1
Ir	0.2 ± 0.1	2 ± 1
Pb*	4.5 ± 1.7	1.3 ± 0.7
B	15 ± 9	36 ± 31
Na	65 ± 26	59 ± 55
Mg*	118 ± 36	32 ± 22
Al	113 ± 5	73 ± 54

The statistical analysis was done using t-test based on a 95% confidence level. Within a given row, an asterisk indicates that the two numbers are significantly different (p < 0.05)

*BDL* below detection limit (K: 0.006, Y: 0.00002, Cd: 0.00006)

#### Lipid Hydrolysis

**Fig. 2:**
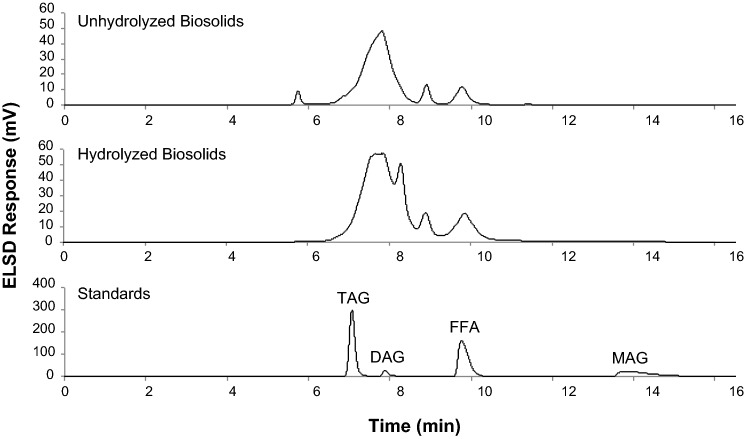
HPLC-ELSD chromatograms of the hexane extractables obtained from biosolids before and after hydrolysis. *TAG* triacylglycerol standard (glyceryl trioleate), *DAG* diacylglycerol standard (glyceryl dioleate), *MAG* monoacylglycerol standard (glyceryl mono-oleate), *FFA* free fatty acid standard (oleic acid). Hydrolysis was performed at 280 °C for 1 h

For integration of biosolids into lipid pyrolysis, a better knowledge of the different classes of lipids present in the biosolids as well as an understanding of the hydrolysis of the lipids contained in biosolids is required. To this end, we employed a well-established HPLC method to separate the various groups of acylglycerols and free fatty acids as shown in Fig. 2. An increase in the free fatty acids is observed after hydrolysis as previously discussed in section "Characterization of Biosolids", with a concurrent decrease in the triacylglycerol peak, suggesting the triacylglycerols are being hydrolyzed to free fatty acids and glycerol. What is evident in Fig. 2 is the relatively large amount of compounds around the diacylglycerol molecular weight range in the hexane extracts of the original biosolids and the hydrolyzed biosolids. The molecular weights of these compounds remained largely unchanged after hydrolysis of biosolids. Based on previously reported work on the hydrolysis of triacylglycerol-based lipids [9], the conditions employed in the hydrolysis in this study was sufficient to convert acylglycerols to free fatty acids. Thus, a large number of compounds observed in the diacylglycerol molecular weight region are non-acylglycerol-based compounds. This further supports the observations discussed in section "Elemental and Metal Analyses" regarding low amount of acylglycerols and free fatty acids in the hexane extract of hydrolyzed biosolids (Table 2).

**Table 2:** CHNS analysis was performed on brown grease, or the lipids fraction from thermal hydrolysis of brown grease with water or biosolids

	Weight % of sample				
	C	H	N	S	O
Brown grease	75.3 ± 0.1	11.6 ± 0.1	< 0.1	< 0.1	11.6 ± 0.2
Water-hydrolyzed Brown grease	76.4 ± 0.1	11.9 ± 0.1	< 0.1	< 0.1	10.7 ± 1.2
Biosolids-hydrolyzed brown grease	76.6 ± 0.1	12.0 ± 0.0	0.4 ± 0.1	< 0.1	11.7 ± 0.1

The analyses were performed in triplicate

**Fig. 3:**
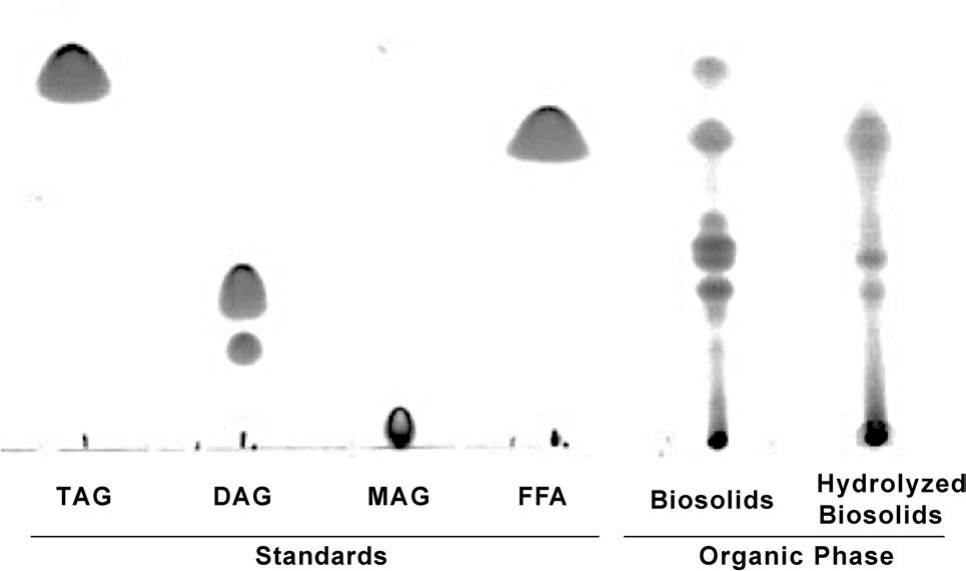
Thin layer chromatogram of the organic phases obtained from hexane extraction of biosolids and hydrolysates. *TAG* triacylglycerol standard (glyceryl trioleate), *DAG* diacylglycerol standard (glyceryl dioleate), *MAG* monoacylglycerol standard (glyceryl mono-oleate), *FFA* free fatty acid standard (oleic acid). Hydrolysis was performed at 280 °C for 1 h

Additionally, thin layer chromatography was also used to rapidly separate based on polarity the different hexane extract obtained from biosolids and hydrolyzed biosolids (Fig. 3). For these experiments, the standards used were pure model triacylglycerol, diacylglycerol, monoacylglycerol, and fatty acids.

As observed in Fig. 3, several different lipid classes were present in the hexane extract of both biosolids and hydrolyzed biosolids. Based on a comparison between the two biosolids samples, it was observed that thermal hydrolysis led to the disappearance of triacylglycerol and some of the diacylglycerol, with a corresponding increase of free fatty acids. This confirms our earlier observation ("Characterization of Biosolids" section) that biosolids contain a significant amount of acylglycerols (as compared to free fatty acids) that can be converted to free fatty acids through thermal hydrolysis.

#### Fatty Acid Profile

**Fig. 4:**
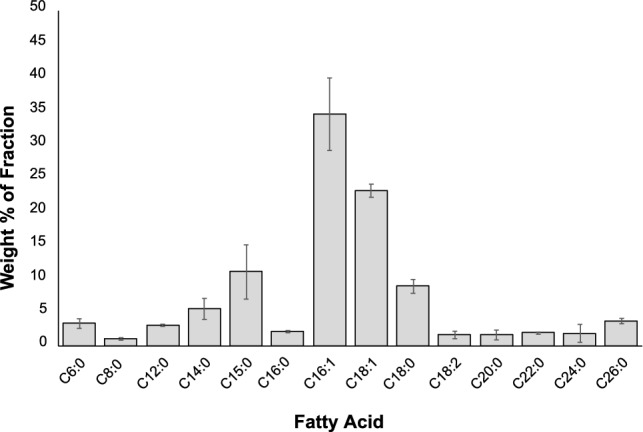
Free fatty acid profile of the organic fraction obtained through hexane extraction of thermally hydrolyzed biosolids. Data shows mean ± standard deviation with n = 3. The amount of each free fatty acid was determined through GC–MS/FID

Even though the levels of fatty acids contained in hydrolyzed biosolids are very low (0.03 ± 0.01%), the fatty acid profile of the hexane extract isolated from biosolids hydrolysates was determined to establish the identity and quantity of fatty acids originating from biosolids (Fig. 4). The fatty acids detected ranged from C6 to C26, with the vast majority being monounsaturated (58 ± 5%) or saturated (41 ± 6%). The two most abundant fatty acids were monounsaturated C16:1 and C18:1, which accounted for 34 ± 5% and 23 ± 1%, respectively, of the total fatty acids. It is worth noting that there was a substantial amount of long chain fatty acids in the range of C20:0 to C26:0 (~ 9.1%), which are excellent precursors to hydrocarbon fuels.

### Hydrolysis of Brown Grease Using Biosolids

#### Biosolids as a Water Replacement

**Fig. 5:**
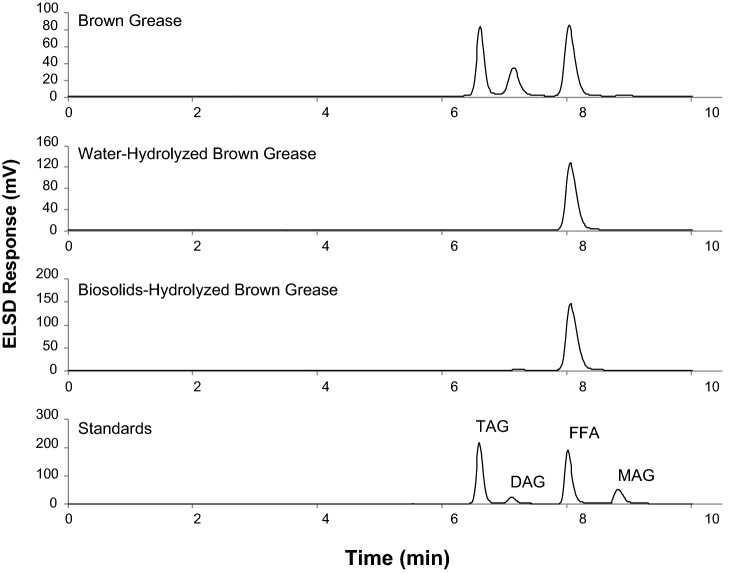
HPLC-ELSD chromatograms of brown grease, or the lipid fraction obtained from brown grease hydrolyzed with water or biosolids. *TAG* triacylglycerol standard (glyceryl trioleate), *DAG* diacylglycerol standard (glyceryl dioleate), *MAG* monoacylglycerol standard (glyceryl mono-oleate), *FFA* free fatty acid standard (oleic acid). Hydrolysis was performed at 280 °C for 1 h

Since the digested biosolids that are found in storage lagoons at wastewater treatment facilities around the world are composed of roughly 93–99% water [7], we investigated the possibility of using biosolids as a water replacement during hydrolysis of brown grease. Hydrolysis of brown grease was performed using either water or biosolids and the results were compared. The hydrolysis was conducted at 280 °C for 1 h at a liquid to lipid mass ratio of 1:1, which was identical to the conditions employed by Asomaning et al. [9]. Figure 5 shows the HPLC-ELSD chromatograms of brown grease, or the lipid fraction obtained after hydrolyzing brown grease and water or biosolids. The chromatograms clearly show the successful conversion of acylglycerols (TAG and DAG) in the brown grease to free fatty acids after hydrolysis with water as well as biosolids. Also evident in Fig. 5 is the absence of the large number of compounds in the diacylglycerol molecular weight range that was present in the hexane extract of hydrolyzed biosolids. This supports our hypothesis that the organic compounds present in the biosolids will not be a significant contributor to the total lipids when biosolids are used as water replacement in the hydrolysis of acylglycerol-based lipids.

**Fig. 6:**
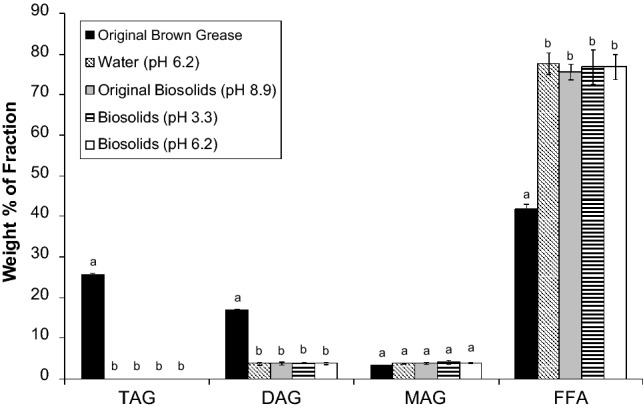
Lipid composition of the organic phase of hydrolyzed brown grease. Hydrolysis of the various systems was conducted at 280 °C for 1 h with a water (or biosolids) to lipid ratio of 1:1. *TAG* triacylglycerol, *DAG* diacylglycerol, *MAG* monoacylglycerol, *FFA* free fatty acids. The pH of the system prior to hydrolysis is shown. Data shows mean ± standard deviation with n = 3. Within a given subclass of lipid (TAG, DAG, MAG, and FFA), mean values with a different letter are statistically different. The statistical analysis was done using one-way ANOVA with mean comparison by Tukey test based on a 95% confidence level

The degrees of hydrolysis obtained using water and biosolids were statistically similar: 99.3 ± 2.2% for water and 98.6 ± 3.9% for biosolids. Furthermore, as shown in Fig. 6, there were no significant differences between the amount of triacylglycerols, diacylglycerols, monoacylglycerols and free fatty acids contained in the organic fraction obtained from hydrolysates derived using water and biosolids. This is a significant finding as based on these data, biosolids can potentially function as a water replacement for the hydrolysis of lipids for biofuels applications. Thus, the findings have far reaching benefits regarding process economics for biofuels production from lipids, particularly waste lipids, while at the same time presenting a potential solution for the problem faced by many municipalities concerning biosolids disposal.

#### Free Fatty Acid Profile of Brown Grease Hydrolysates

**Fig. 7:**
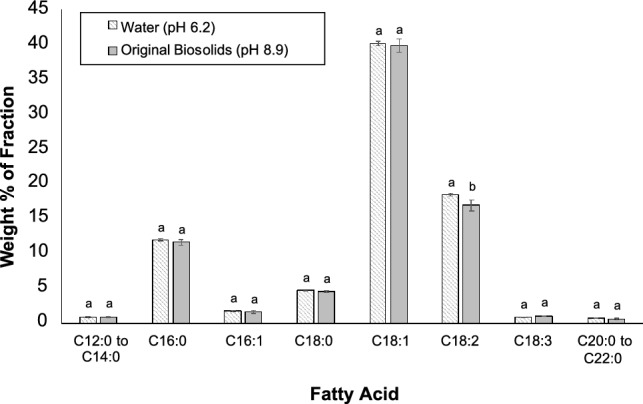
Free fatty acid distribution in the organic phase obtained from brown grease hydrolyzed with either water or biosolids. Brown grease was hydrolyzed at 280 °C for 1 h at a liquid to lipid ratio of 1:1. Data shows mean ± standard deviation with n = 3. The statistical analysis was done using t-test based on a 95% confidence level. For a given fatty acid, mean values with a different letter are statistically different

To investigate whether any of the components in biosolids could alter the fatty acid profile of the brown grease hydrolysates, we compared the composition of fatty acids in the lipid fractions from hydrolysis of brown grease derived using water or biosolids (Fig. 7). Since diesel fuel generally consists of hydrocarbons with a chain length of 12–20 carbon atoms, medium and long chain fatty acids are generally preferred for biofuel applications particularly, renewable diesel. Other than linoleic acid (C18:2), there were no significant differences in the amount of fatty acids present in the lipid fraction when water or biosolids were used for hydrolysis of brown grease. Even for linoleic acid, the amounts observed in the two systems was comparable: 18.4 ± 0.2% versus 16.9 ± 0.8% when water and biosolids were used, respectively. In both systems, oleic and linoleic acids were the two most abundant fatty acids, comprising almost 60% of the lipid fraction after hydrolysis. Palmitic and stearic acids were the most abundant saturated fatty acids, accounting for ~ 12% and ~ 5% of the organic phase, respectively. Based on these data, the fatty acid profile obtained through hydrolysis of brown grease with biosolids is comparable to that observed when water was used, which further supports the use of biosolids as a water replacement during the hydrolysis step.

Although the use of biosolids rather than water for the hydrolysis of brown grease did not have an effect on the degree of hydrolysis or lipid composition, there are other factors that need to be considered when incorporating biosolids into fuel processing strategies. For example, sewage sludge and biosolids are known to contain sulfolipids and phospholipids [18, 19]. If these lipids are carried forward into the pyrolysis reaction, there is a risk that some sulphur and phosphorous may be carried into the final fuel products. The levels of sulphur and phosphorous in fuels are tightly regulated; upon combustion, sulphur forms gaseous oxides (SO_x_) that promote production of acid rain [20], while phosphorous is known to damage car engines [21]. Thus, it will be necessary to ascertain how the presence of even a small amount of sulpholipids and phospholipids in the organic phase impacts the quality of fuel generated through pyrolysis of fatty acids.

#### The Effect of pH on Hydrolysis of Brown Grease with Biosolids

Previous work in our group established that thermal hydrolysis improved the settling rates of biosolids, and that this phenomenon intensified when the pH was lowered to 3 [8]. Consequently, we performed experiments to establish whether thermal hydrolysis of brown grease with biosolids at different pH impacted the degree of lipid hydrolysis. Although it is well established that hydrolysis of fats and lipids can occur through either acid- or base-catalyzed reactions, albeit through different mechanisms [22, 23], it is possible that these reactions could be influenced by chemicals and/or molecules found in biosolids.

Lowering the pH of the brown grease/biosolids mixture to 6.2 (the pH of the distilled water system) or 3.3 did not have a significant impact on the degree of hydrolysis (92.7 ± 3.7 and 94.6 ± 1.0, respectively). These values were statistically similar to each other, as well as to the degrees of hydrolysis achieved using water or biosolids (pH 8.9), which were presented in section "Characterization of Biosolids". Furthermore, in the two systems where the pH was adjusted prior to thermal treatment, the levels of triacylglycerols, diacylglycerols, monoacylglycerols, and free fatty acids were statistically similar to the system where biosolids were used without pH adjustment, as well as the system where water was used for hydrolysis (Fig. 6). Thus, in terms of the degree of hydrolysis and the lipid profile following hydrolysis, lowering the pH of the system did not have a significant impact.

#### Settling of Biosolids

**Fig. 8:**
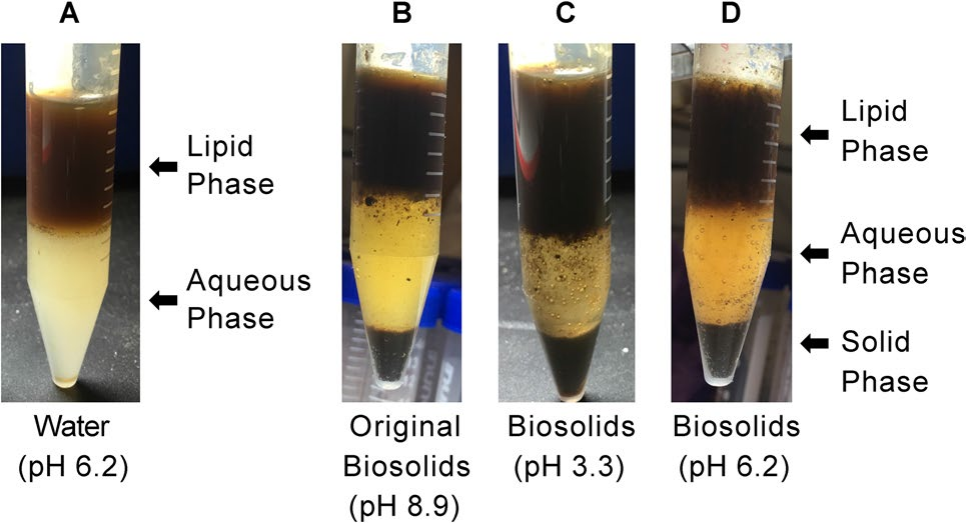
Hydrolysates from hydrolysis of brown grease with water (**a**) or biosolids (**b**–**d**). For the systems incorporating biosolids, the brown grease/biosolids mixture was used as is (pH 8.9; **b**), or adjusted to pH 3.3 (**c**) or 6.2 (**d**) prior to hydrolysis. Hydrolysis was performed at 280 °C for 1 h at liquid to lipid mass ratio of 1:1

Chae et al. [8] demonstrated that thermal hydrolysis of biosolids resulted in significantly better settling rates of suspended solids, particularly when the pH was adjusted to 3 prior to thermal treatment. Similarly, in all three systems where biosolids were used to hydrolyze brown grease, a solid fraction was observed that was not observed in the hydrolysate where distilled water was employed for hydrolysis (Fig. 8). The solid fraction, aqueous phase, and organic phase could be easily separated through natural settling in a matter of minutes. This was true regardless of the pH of the system, although the precise settling rates were not measured in these experiments. These data suggest that when biosolids are used for hydrolysis of brown grease, it not only serves as a water replacement and a source of a small amount of lipid material, but can also function to dramatically improve settling rates of biosolids. Thus, incorporation of biosolids in lipid pyrolysis may not only benefit the biofuels industry, but could also serve as a promising strategy for the disposal of increasing amounts of biosolids worldwide.

#### Effect of Hydrolysis Temperature

Thermal treatments such as hydrolysis are associated with high operation costs that could be a barrier to commercialization. To help address this concern, we examined whether thermal hydrolysis of brown grease could be conducted at a lower temperature than that established by Asomaning et al. [9]. Thermal hydrolysis of brown grease with water and biosolids at 260 °C resulted in degrees of hydrolysis of 96.2 ± 0.5% and 94.5 ± 2.4%, respectively, which were statistically similar to the reactions performed at 280 °C with either water or biosolids (described in section "Characterization of Biosolids"). These data suggested that lowering the temperature of thermal hydrolysis to 260 °C does not impact the conversion of acylglycerol-based lipids to free fatty acids. The potential cost savings provided by employing a lower temperature for thermal hydrolysis may help to improve process economics.

Thermal hydrolysis is already a commonly used method in wastewater treatment facilities, though it is typically used to treat primary sewage sludge, not digested biosolids. The temperature generally employed for thermal hydrolysis of primary sludge is in the range of 165–180 °C as higher temperatures have been shown to lead to decreased rates of natural gas formation in subsequent digestion steps [24–26]. However, the digestibility of brown grease/biosolids hydrolysates is not a concern for lipid pyrolysis and thus higher temperatures can be employed. It should be noted that Wilson and Novak [27] examined different hydrolysis temperatures (130–220 °C) for treatment of primary sewage sludge and observed that lipid hydrolysis improved with increasing temperature and increasing degrees of unsaturation. Thus, the thermal hydrolysis temperature employed for hydrolysis of brown grease will likely need to be higher than what is commonly used in the literature for treatment of sewage sludge to ensure maximum efficiency. Since reducing the temperature of thermal hydrolysis in our experiments from 280 to 260 °C did not impact the degree of lipid hydrolysis, an optimization study should be performed to establish the conditions required for maximum hydrolysis of brown grease with biosolids.

## Conclusion

The research presented above provides proof-of-concept that biosolids from wastewater treatment lagoons can be used as a water replacement during the hydrolysis of acylglycerol-based lipids. At the same time, the high temperatures employed for hydrolysis of brown grease can dramatically improve settling rates of biosolids. Thus, integration of biosolids into lipid pyrolysis may lower costs associated with fuel production while also offering a mechanism for disposal of the increasing amounts of biosolids that are anticipated worldwide. Successful application of this approach could have far reaching impacts on both the biofuel and waste management sectors. Some final questions remain on the impact of sulphur content and trace minerals that will require further investigation prior to commercial evaluation of the approach.
